# Case Report: Electrode perforation with normal pacemaker parameters leading to hemorrhagic shock: a rare case report of the “penetration-exit-re-entry” phenomenon

**DOI:** 10.3389/fmed.2026.1943190

**Published:** 2026-09-03

**Authors:** Zuoan Qin, Jian Yang, Li Luo, Shi Chen, Fei Yang

**Affiliations:** 1 Department of Cardiology, Changde Hospital, Xiangya School of Medicine, Central South University (The First People’s Hospital of Changde City), Changde, China; 2 Department of Cardiac Catheterization Laboratory, Changde Hospital, Xiangya School of Medicine, Central South University (The First People’s Hospital of Changde City), Changde, China; 3 Department of Chest Surgery, Changde Hospital, Xiangya School of Medicine, Central South University (The First People’s Hospital of Changde City), Changde, China

**Keywords:** dual-chamber pacemaker, hemorrhagic shock, lead penetration-exit-re-entry injury, massive hemothorax, subclavian vein rupture

## Abstract

**Background:**

Permanent pacemaker implantation via subclavian vein puncture may induce rare but life-threatening iatrogenic vascular injury. Vascular perforation with pacing lead penetration-exit-re-entry trajectory accompanied by normal intraoperative pacing parameters is reported extremely rarely, which greatly increases the risk of missed diagnosis.

**Case presentation:**

A 70-year-old female patient with complete atrioventricular block received dual-chamber pacemaker implantation. All intraoperative lead electrical indicators were within normal range. Immediately after the operation, she rapidly developed massive hemothorax and hemorrhagic shock with severe hypoxemia. A thoracoscopic exploration verified a unique “penetration-exit-re-entry” lesion: the pacing electrode penetrated the left subclavian vein wall, exited the vascular lumen, and re-entered the innominate vein, resulting in persistent intrathoracic bleeding. After emergency thoracic drainage, surgical venous repair and blood transfusion resuscitation, the patient's vital signs gradually stabilized and fully recovered. A 1-month follow-up showed stable pacemaker function without adverse events.

**Conclusion:**

This case highlights that normal intraoperative pacing parameters cannot rule out occult extravascular lead perforation and venous laceration. Operators should maintain high vigilance for unexplained perioperative hemodynamic collapse. Combined imaging, diagnostic thoracentesis, and early thoracoscopic surgical intervention are the core rescue strategies for this fatal complication.

## 1 Introduction

Permanent pacemaker implantation is a mature intervention for symptomatic bradyarrhythmias, including sick sinus syndrome and atrioventricular block (1). Despite its overall favorable safety profile, multiple perioperative complications may occur, such as pneumothorax, hemothorax, iatrogenic vascular tear, cardiac perforation, lead dislodgement, and surgical site infection (2, 3). The subclavian vein remains the most widely adopted puncture access for pacing lead delivery because of its superficial anatomical location and convenient operative approach (4).

Among all procedural adverse events, venous wall perforation induced by guidewires or pacing electrodes can trigger concealed hemorrhage, rapid-onset massive hemothorax, and progressive hemorrhagic shock. This complication carries low incidence but extremely high mortality, and its insidious clinical manifestations often lead to delayed diagnosis (5).

Most previously reported pacemaker-related hemothorax cases presented abnormal pacing thresholds or lead dislocation intraoperatively. However, we herein report a rare case of a patient in whom the pacing electrode formed a special “penetration-exit-re-entry” path across the left subclavian vein–innominate vein junction, with completely normal intraoperative electrical parameters, followed by acute life-threatening hemorrhagic shock. This case report aims to raise clinician awareness of this easily missed severe complication and summarize standardized early identification and multidisciplinary collaborative management strategies referenced to current clinical evidence.

## 2 Case presentation

A 70-year-old female patient was admitted with a 3-year history of transient syncope and a 1-month history of exertional dyspnea, fatigue, chest tightness, and palpitations. Her past medical history included 10 years of poorly controlled hypertension (peak systolic blood pressure >200 mmHg, treated with the nifedipine gastrointestinal therapeutic system and terazosin) and stage 4 chronic renal failure.

### 2.1 Preoperative assessment

Vital signs on admission were as follows: body temperature 36.5°C, heart rate 46 beats per minute, respiratory rate 18 breaths per minute, and blood pressure 233/116 mmHg. A cardiac auscultation revealed regular bradycardia at 46 bpm without pathological murmurs. Laboratory examinations produced the following results: serum creatinine 483.5 μmol/L, hemoglobin 72 g/L (moderate anemia), and D-dimer 1.25 μg/mL. A 24-h Holter monitoring confirmed sinus bradycardia (mean heart rate 49 bpm), frequent bifocal ventricular premature beats, junctional escape rhythm, complete atrioventricular block, and diffuse ST-T segment abnormalities. Preoperative composite diagnoses included complete atrioventricular block, NYHA Class III heart failure, grade 3 very high-risk hypertension, stage 4 chronic kidney disease, and moderate anemia.

### 2.2 Periprocedural antithrombotic management

The patient was not receiving long-term anticoagulant or antiplatelet therapy. Because of her moderate anemia (hemoglobin 72 g/L) and anticipated periprocedural bleeding risk, systemic antithrombotic prophylaxis was withheld before and during the procedure.

### 2.3 Pacemaker implantation and intraoperative abnormality

On hospital day 5, a dual-chamber permanent pacemaker implantation was performed under local anesthesia. Vascular access was obtained by conventional anatomic landmark–guided left subclavian vein puncture; neither real-time ultrasound guidance nor contrast venography was used. The puncture site was positioned ∼2 cm lateral to the left sternoclavicular joint, directly inferior to the clavicle and superficial to the first rib. Following two successful venipunctures, guidewires were advanced to the inferior vena cava under fluoroscopic surveillance. Then, 7-French peel-away sheaths were inserted, with sheath tips positioned within the proximal left subclavian vein, immediately upstream of the innominate vein confluence prior to lead deployment. An active-fixation ventricular lead (Model 3830, Medtronic) was advanced to the left bundle branch region of the right ventricular interventricular septum, with a pacing threshold of 1.0 V at 0.42 ms, an R-wave sensing amplitude of 7.0 mV, and a lead impedance of 640 Ω. An atrial active-fixation lead (Medtronic 4574-53) was anchored at the right atrial appendage, with a pacing threshold of 1.0 V at 0.42 ms, a P-wave sensing amplitude of 2.0 mV, and a lead impedance of 600 Ω. All leads were connected to a dual-chamber pulse generator (Medtronic EN1DR01) and fixed, followed by routine incision suturing.

An intraoperative fluoroscopy confirmed normal guidewire and lead courses toward the inferior vena cava and right heart; neither pleural effusion nor mediastinal widening was apparent during the procedure. During advancement of the ventricular lead through the sheath, the patient suddenly complained of severe sharp left chest pain and progressive dyspnea; at that moment, blood pressure rose to 190/100 mmHg. The symptoms were initially attributed to pain-induced acute left heart failure, and intravenous furosemide (20 mg), morphine (3 mg), and sodium nitroprusside were administered for symptomatic relief.

### 2.4 Intraoperative hemodynamic course

Continuous invasive arterial blood pressure monitoring was not established before the crisis. Non-invasive cuff measurements were recorded at approximately 10-min intervals by using the automated anesthesia monitor and are summarized in Table 1. Briefly, blood pressure before sedation was 233/116 mmHg. After the first successful venipuncture and sheath insertion, blood pressure was approximately 180/96 mmHg. At the onset of chest pain (during ventricular lead advancement), blood pressure increased to 190/100 mmHg. Immediately after wound closure, the patient became cold and clammy; blood pressure fell precipitously from 190/100 to 60/40 mmHg within a single measurement interval, and peripheral oxygen saturation dropped to 60%, prompting emergency endotracheal intubation, mechanical ventilation, and vasopressor support.

**Table 1:** Serial non-invasive hemodynamic measurements during pacemaker implantation.

Time point	Blood pressure (mmHg)	Heart rate (bpm)	SpO2 (%)
Before sedation	233/116	46	94
After first venipuncture/sheath insertion	∼180/96	∼52	95
During ventricular lead advancement (onset of pain)	190/100	∼58	93
Immediately after wound closure	60/40	∼110	60

### 2.5 Acute postoperative hemorrhagic shock and diagnostic workup

Immediately after the surgery, the patient developed cold sweats, altered mental status, and severe hypotension (60/40 mmHg; sodium nitroprusside was discontinued immediately), and peripheral oxygen saturation dropped to 60%. Emergency volume resuscitation and vasopressor support (dopamine, metaraminol) were initiated; anesthesiologists performed endotracheal intubation and invasive mechanical ventilation.

**Figure 1:**
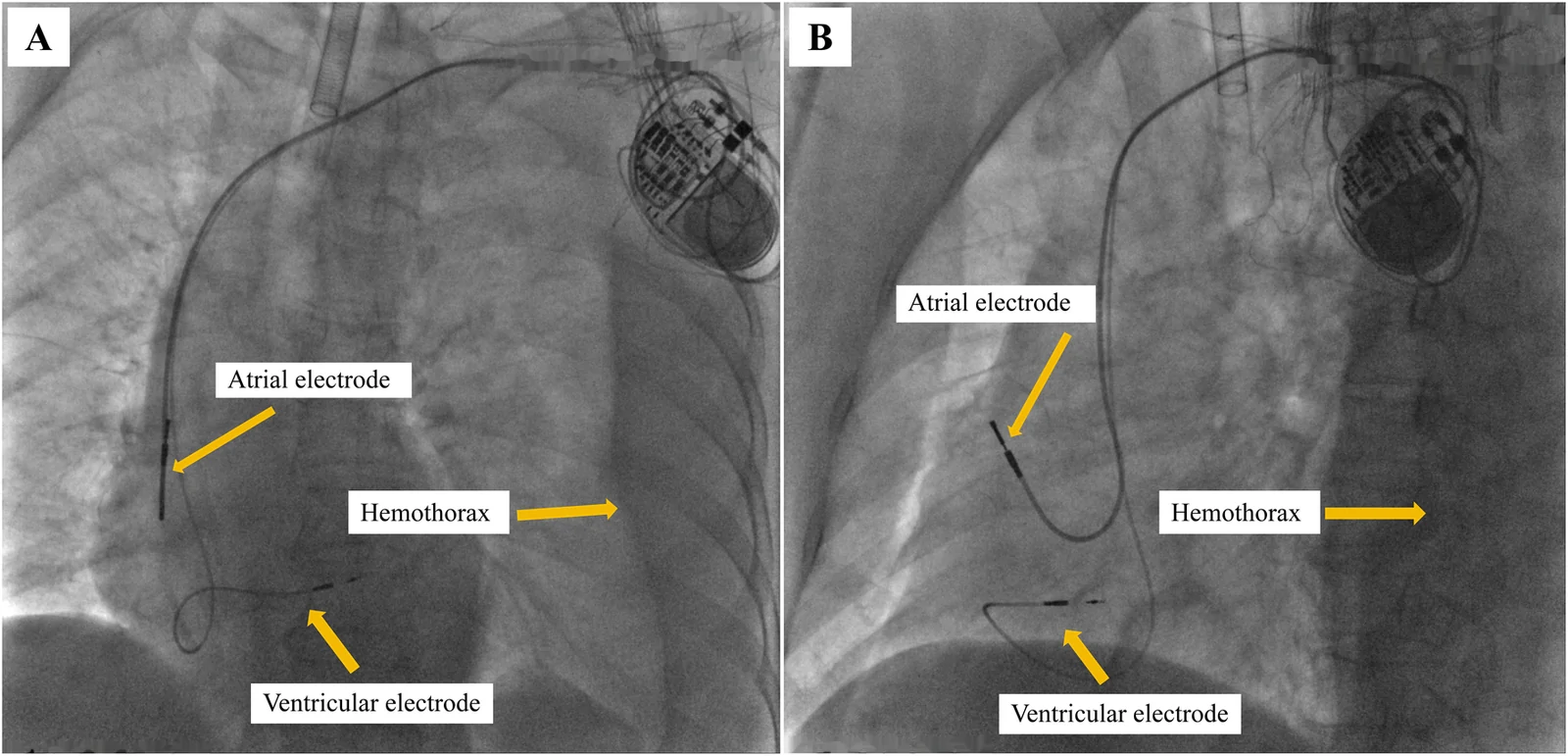
Anteroposterior **(A)** and 45° left anterior oblique **(B)** intraprocedural fluoroscopic radiographs demonstrating diffuse homogeneous opacification of the entire left hemithorax, consistent with severe postprocedural hemothorax. The modified radiograph excludes overlay cardiac device graphics to provide unobscured visualization of the left clavicular region, subclavian venous puncture site, proximal lead trajectory, and osseous landmarks. The labeled yellow arrows denote the atrial lead and ventricular lead, respectively.

**Figure 2:**
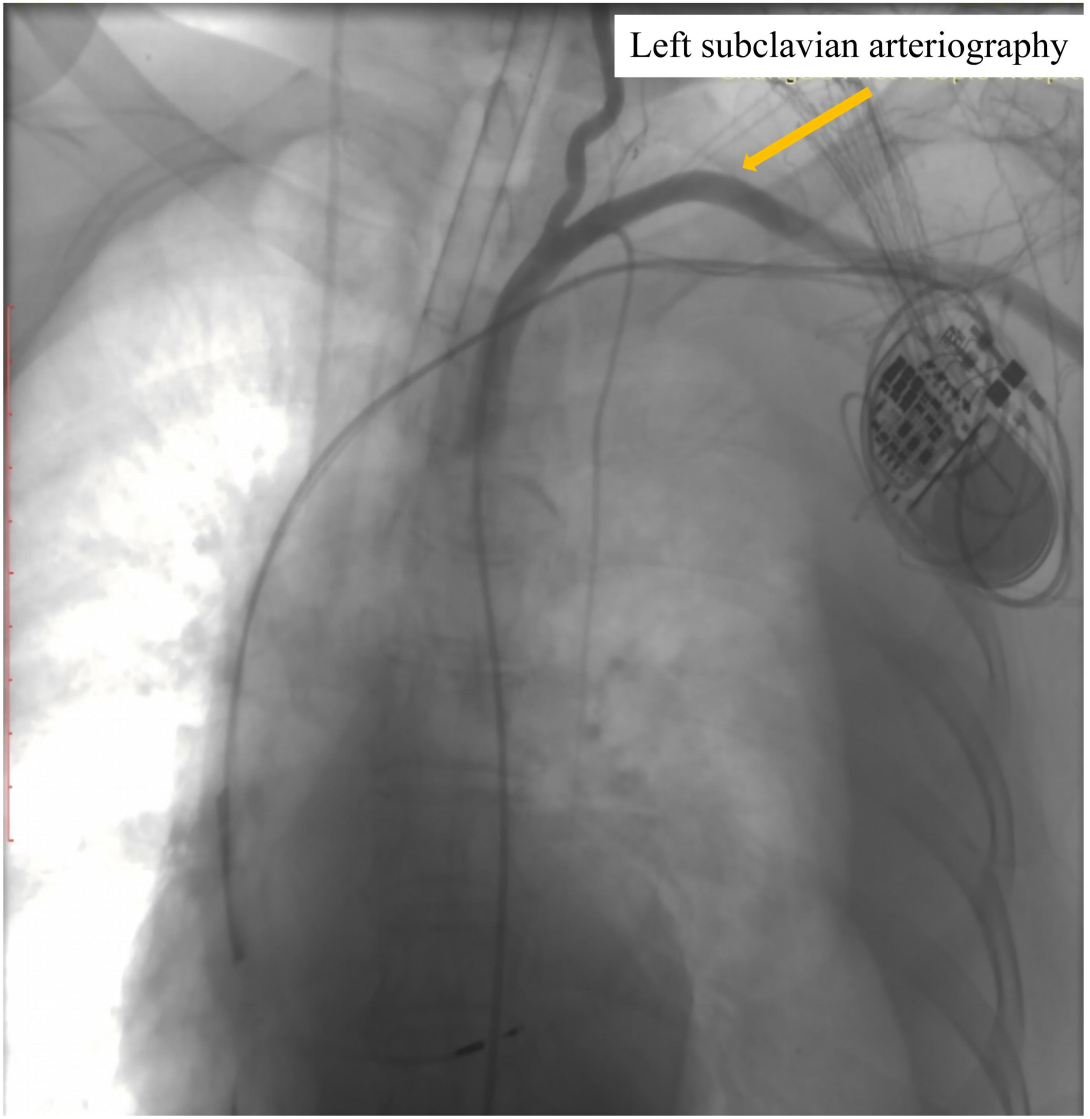
Left subclavian arteriography via femoral artery access, demonstrating an intact arterial vessel wall without extravasation of the contrast agent, excluding the arterial bleeding source. The yellow arrow marks the left subclavian artery trunk.

A postprocedural fluoroscopy (anteroposterior view + 45° left anterior oblique view) demonstrated extensive homogeneous opacification occupying the whole left thoracic cavity with obscured pulmonary margins, consistent with massive hemothorax (Figure 1). Femoral artery access via the left subclavian artery and aortic arch angiography excluded arterial rupture and bleeding source (Figure 2). Diagnostic thoracentesis yielded non-clotting bloody pleural fluid, confirming traumatic hemothorax after multidisciplinary consultation with the thoracic surgery team.

### 2.6 Emergency surgical intervention and postoperative recovery

**Figure 3:**
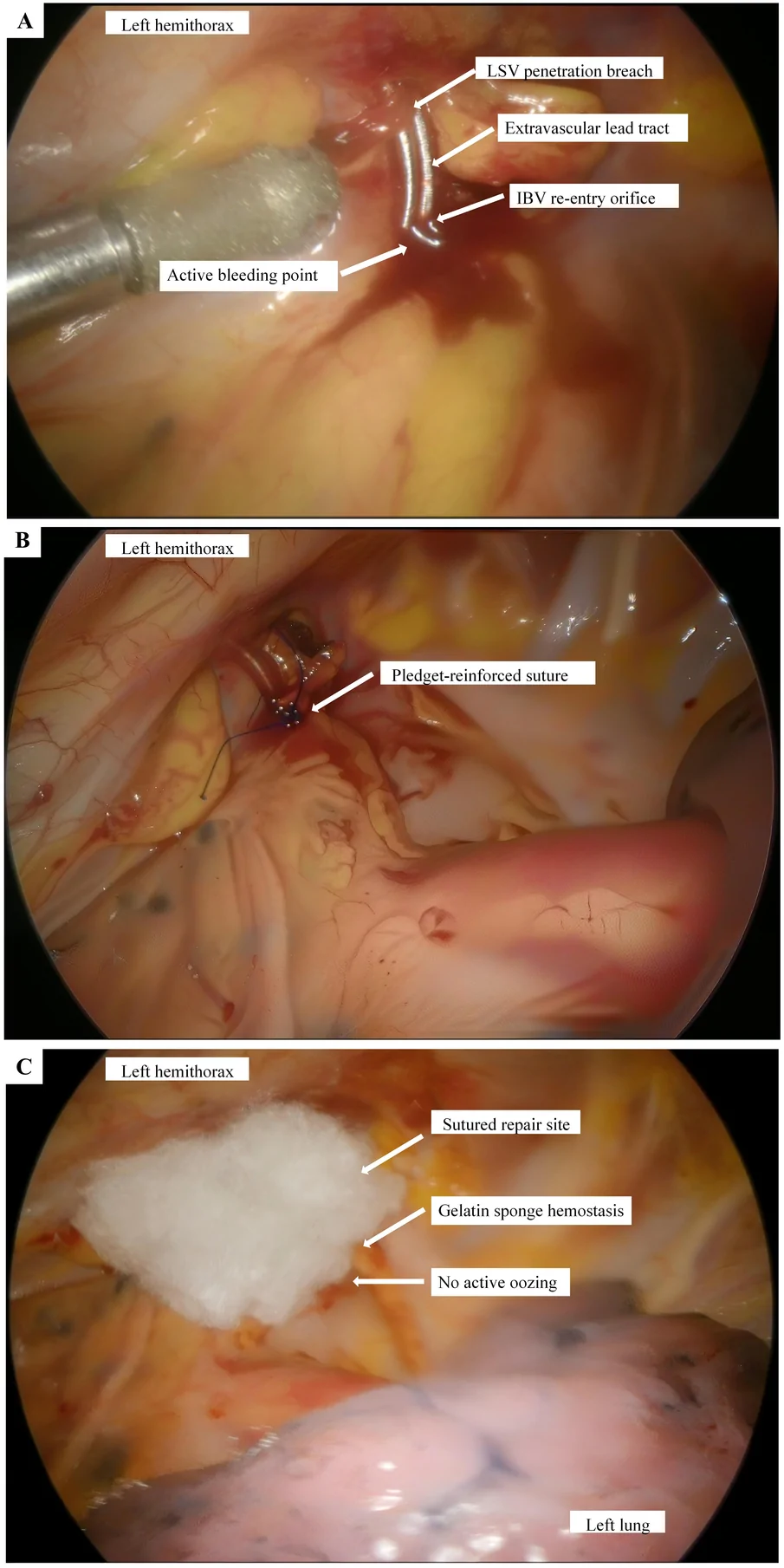
Serial video-assisted thoracoscopic surgery (VATS) images captured during exploration and staged surgical repair of the unique penetration-exit-re-entry venous injury, with standardized anatomical orientation labels for enhanced interpretability. All panels display the left hemithorax at the level of the thoracic inlet mediastinum. **(A)** Initial thoracoscopic exploration revealing the full penetration-exit-re-entry trajectory of the pacing lead. Annotated landmarks: LSV penetration breach (primary venous tear site); extravascular lead tract extending outside the venous lumen; IBV re-entry orifice where the lead re-enters the brachiocephalic venous system; focal active intrathoracic hemorrhage originating from the LSV laceration. **(B)** Intraoperative repair phase showing pledget-reinforced interrupted sutures placed to close the LSV venous laceration defect under thoracoscopic visualization. **(C)** Final hemostatic assessment after surgical repair: the sutured venous repair site is covered with absorbable gelatin sponge for local compressive hemostasis, with no residual active bleeding or tissue oozing identified. LSV, left subclavian vein; IBV, innominate (brachiocephalic) vein; VATS, video-assisted thoracoscopic surgery.

An emergency surgical intervention in the form of closed thoracic drainage was performed by inserting a 24-French chest tube into the left posterior axillary line fifth intercostal space, which yielded an initial drainage volume of 1200 mL dark blood. The patient's blood pressure slightly improved but remained unstable. A subsequent thoracoscopic exploration was conducted through a 6 cm posterolateral incision at the left third intercostal space. Severe intrathoracic adhesions were observed, and approximately 600 mL accumulated blood and clots were evacuated. An intraoperative thoracoscopic vision confirmed that the pacing electrode penetrated the left subclavian vein wall, exited the vascular lumen, and re-entered the innominate vein, causing continuous active bleeding (Figure 3A). The venous laceration was repaired with pledget-reinforced interrupted sutures (Figure 3B). After gelatin sponge compression hemostasis, no active oozing was observed (Figure 3C). A thoracic drainage tube was retained, and the thoracic cavity was closed in layers. The intraoperative blood loss totaled ∼50 mL; 2 units of packed red blood cells were transfused perioperatively.

The patient was transferred to the cardiac care unit for comprehensive critical care, including blood transfusion, vasoactive agent titration, regular hemodialysis, and broad-spectrum antimicrobial prophylaxis. Her vital signs gradually stabilized, and vasopressors were tapered stepwise. Endotracheal extubation was completed on postoperative day 2. Daily drainage volume declined progressively, and the chest tube was removed on postoperative day 5. A follow-up chest radiography showed no residual massive left pleural effusion; a routine electrocardiography verified normal pacemaker capture and sensing function. The patient achieved full clinical recovery and was discharged on postoperative day 20. At the 1-month outpatient follow-up, the patient remained asymptomatic with stable pacing electrical parameters.

## 3 Discussion

Subclavian vein puncture represents the standard vascular access for cardiac pacing device implantation, while iatrogenic venous injury remains an unavoidable potential procedural complication. Published literature reports overall subclavian puncture-related complication incidence ranging from 1% to 5%, among which hemothorax and pneumothorax account for 0.5%–2% (5, 6). Nevertheless, combined vascular lesion featuring pacing lead penetration of the subclavian vein, extravascular exit, re-entry into the innominate vein, persistent venous bleeding, and subsequent acute massive hemothorax are all extremely rare in clinical practice.

### 3.1 Mechanism of the “penetration-exit-re-entry” vascular injury

Excessive medial angulation of puncture needles or forced advancement of resistance-bearing guidewires during left subclavian vein cannulation is the primary trigger for venous wall perforation into the thoracic cavity. Subsequent delivery of vascular sheaths and pacing electrodes further enlarges the venous tear site. In the present patient case, the primary lesion was a venous laceration at the left subclavian vein–innominate vein junction. The electrode penetrated the vein wall at this laceration, exited the vascular lumen, coursed through the mediastinal tissues, and re-entered the innominate vein, thereby reaching standard intracardiac positions. Accordingly, the term “penetration-exit-re-entry” characterizes the lead's extravascular course, whereas the clinically significant lesion is the venous wall laceration. Intraprocedural fluoroscopy failed to identify early hemothorax, which became radiographically apparent only on postprocedural imaging after substantial intrathoracic hemorrhage had accumulated.

Notably, all intraoperative electrical indicators of both atrial and ventricular leads remained within normal reference ranges in this patient. This finding was expected because the lead tips were not displaced; it only indicated that the electrode tip reached an intracardiac target, but it did not exclude an extravascular lead segment. This occult vascular laceration therefore cannot be identified via intraprocedural electrical testing, and clinical decompensation emerges only when persistent hemorrhage elevates intrathoracic pressure to a critical threshold. In addition, long-standing uncontrolled hypertension and stage 4 chronic renal failure aggravated vascular wall fragility, amplifying intraoperative bleeding severity.

### 3.2 Differential diagnosis of intraoperative acute hypotension

Sudden perioperative hypotension requires differential diagnosis with multiple lethal critical illnesses, including acute left heart failure, cardiac tamponade, anaphylactic shock, tension pneumothorax, and massive hemothorax. In this case, the patient presented with transient hypertension and received sodium nitroprusside infusion before he experienced severe hypotension, which initially misled clinicians to suspect vasodilator overdose or aggravated heart failure. However, concurrent severe hypoxemia, cold clammy skin, absence of jugular venous distension, and muffled heart sounds (classic cardiac tamponade manifestations), combined with fluoroscopic pleural effusion signs, strongly indicated intrathoracic hemorrhage. Diagnostic thoracentesis served as a rapid, reliable confirmatory tool for hemothorax and contributed decisively to successful resuscitation.

This case highlights that unexplained periprocedural hypotension following subclavian venous cannulation should not be prematurely attributed to the vasodilatory effects of antihypertensive agents. Instead, occult hemothorax must be promptly ruled out via bedside imaging and diagnostic thoracentesis when clinically indicated.

### 3.3 Standardized multidisciplinary clinical management

Once massive hemothorax is confirmed, an emergency closed thoracic drainage must be performed immediately to relieve elevated intrathoracic pressure and quantify total blood loss. Meanwhile, aggressive crystalloid/colloid resuscitation and blood product transfusion are mandatory to maintain systemic circulation stability. If hypotension persists after drainage or hourly drainage volume exceeds 200 mL, active intrathoracic vascular bleeding is highly suspected, and emergency surgical exploration is indicated without delay. In this case, a femoral artery angiography excluded arterial hemorrhage source. Timely joint intervention by cardiologists and thoracic surgeons via thoracoscopic surgery successfully localized and repaired the venous rupture site, which was the decisive factor for patient survival. Existing clinical studies confirm that conservative treatment carries extremely high failure rates for subclavian–innominate vein junction laceration, and surgical repair is the only definitive therapeutic option (7, 8).

Compared with previously published pacemaker-associated hemothorax case series (9–11), the present case possesses three distinctive clinical characteristics: (1) vascular injury localized at the anatomical junction of the left subclavian vein and innominate vein; (2) pacing lead regained normal intracardiac positioning after extravascular penetration, leading to fully normal intraoperative electrical parameters and significantly increasing missed diagnosis risk; and (3) total estimated intrathoracic blood loss exceeded 1,800 mL, triggering rapid progression to refractory hemorrhagic shock. This case fully demonstrates that normal intraprocedural pacing lead electrical parameters cannot completely exclude concealed extravascular vascular injury.

### 3.4 Preventive strategies for intraoperative vascular perforation

Combined with this critical case and relevant clinical evidence, we summarize standardized preventive recommendations for pacing device implantation:

1. Avoid excessive medial deviation or oversteep angulation during subclavian vein puncture; advance guidewires gently without forced manipulation against obvious resistance.
2. Maintain high suspicion of iatrogenic vascular injury if patients complain of unusual local shoulder/chest pain, poor venous blood return, sudden cough, or severe retrosternal discomfort during puncture or guidewire advancement. For high-risk patients—such as the elderly, those with long-standing uncontrolled hypertension, diabetes mellitus, or renal insufficiency, or those with difficult anatomy—we recommend preprocedure venous ultrasound mapping and preferential use of ultrasound-guided axillary vein puncture or contrast venography to confirm venous anatomy before sheath insertion (12, 13). These techniques may reduce the risk of vascular trauma by allowing direct visualization of the vein and avoiding inadvertent deep or medial puncture.
3. Mandate routine intraoperative fluoroscopy to confirm guidewire advances toward the inferior vena cava rather than mediastinum or pleural cavity.
4. Strengthen intraoperative hemodynamic monitoring and gentle manipulation for high-risk populations, including elderly patients, individuals with long-term uncontrolled hypertension, diabetes mellitus, or renal insufficiency.

### 3.5 Institutional protocol changes after this case

In response to this critical adverse event, our institutional clinical protocol for permanent pacemaker implantation was comprehensively revised. The updated protocol mandates routine preprocedural venous ultrasound mapping, prioritizes ultrasound-guided axillary venous access or contrast venography for patients with high-risk clinical profiles, and establishes standardized intraprocedural hemodynamic monitoring with a low threshold for urgent bedside imaging upon the onset of unexplained hypotension or thoracic pain. These practical measures were introduced to reduce the risk of similar occult vascular injuries and to facilitate earlier recognition.

### 3.6 Limitations of this case report

This study carries inherent limitations as a single case report, and its conclusions cannot be generalized to all pacemaker implantation populations. In addition, we lack long-term follow-up data over a 1-year period to observe late vascular complications related to the repaired venous site. Further large-scale retrospective cohort studies are required to quantify the incidence and prognostic factors of this “penetration-exit-re-entry” vascular complication.

## 4 Conclusion

Subclavian vein perforation secondary to guidewire or pacing lead trauma, followed by lead re-entry into the innominate vein, constitutes a rare yet lethal complication of permanent pacemaker implantation, capable of inducing massive hemothorax and rapidly progressive hemorrhagic shock. Operators must maintain heightened vigilance for abnormal procedural resistance and unexplained periprocedural hemodynamic collapse. Expedited multimodal diagnostic workup (including diagnostic thoracentesis, bedside ultrasonography, and fluoroscopy), urgent thoracic decompression via tube thoracostomy, and early video-assisted thoracoscopic venous repair constitute the cornerstone of life-saving management for these critically ill patients.

## Data Availability

The original contributions presented in the study are included in the article/Supplementary Material, further inquiries can be directed to the corresponding authors.
